# Bile acids induce IL-1α and drive NLRP3 inflammasome-independent production of IL-1β in murine dendritic cells

**DOI:** 10.3389/fimmu.2023.1285357

**Published:** 2023-11-27

**Authors:** Ewa Oleszycka, Eoin C. O’Brien, Michael Freeley, Ed C. Lavelle, Aideen Long

**Affiliations:** ^1^ Adjuvant Research Group, School of Biochemistry and Immunology, Trinity Biomedical Sciences Institute, Trinity College Dublin, Dublin, Ireland; ^2^ Department of Immunology, Faculty of Biochemistry, Biophysics and Biotechnology, Jagiellonian University, Kraków, Poland; ^3^ School of Biotechnology, Dublin City University, Dublin, Ireland; ^4^ Department of Clinical Medicine, School of Medicine, Trinity Translational Medicine Institute, Trinity College Dublin, Dublin, Ireland

**Keywords:** bile acids, IL-1α, IL-1β, dendritic cells, inflammation

## Abstract

Bile acids are amphipathic molecules that are synthesized from cholesterol in the liver and facilitate intestinal absorption of lipids and nutrients. They are released into the small intestine upon ingestion of a meal where intestinal bacteria can modify primary into secondary bile acids. Bile acids are cytotoxic at high concentrations and have been associated with inflammatory diseases such as liver inflammation and Barrett’s Oesophagus. Although bile acids induce pro-inflammatory signalling, their role in inducing innate immune cytokines and inflammation has not been fully explored to date. Here we demonstrate that the bile acids, deoxycholic acid (DCA) and chenodeoxycholic acid (CDCA) induce IL-1α and IL-1β secretion *in vitro* in primed bone marrow derived dendritic cells (BMDCs). The secretion of IL-1β was found not to require expression of NLRP3, ASC or caspase-1 activity; we can’t rule out all inflammasomes. Furthermore, DCA and CDCA were shown to induce the recruitment of neutrophils and monocytes to the site of injection an intraperitoneal model of inflammation. This study further underlines a mechanistic role for bile acids in the pathogenesis of inflammatory diseases through stimulating the production of pro-inflammatory cytokines and recruitment of innate immune cells.

## 1 Introduction

IL-1α/β family cytokines are proinflammatory and can induce vasodilation, upregulation of adhesion molecules on endothelial cells and chemokine production facilitating infiltration of immune cells into a site of tissue damage or infection (1). Both, IL-1α and IL-1β bind to the heterodimer receptor IL-1RI-IL-1RAcP, however these cytokines differ in their mode of activation. IL-1α is synthesized in the cell as a 31kDa pre-cursor form (pro-IL-1α) which is biologically active and has the ability to bind to the IL-1 receptor in this state (2). Under certain conditions cleavage of pro-IL-1α can occur which allows for modulation of its activity (3, 4). IL-1α release is often associated with inflammation as a result of necrotic cell death but not seen during apoptosis. In cells undergoing apoptosis, IL-1α is transported to the nucleus to prevent its release into the extracellular space (5). This prevents sterile inflammation occurring as a result of IL-1α release. IL-1α interacts with the IL-1 receptor at the surface of endothelial cells which in turn release proinflammatory molecules that can recruit neutrophils and macrophages, induce fever and activate adaptive immune cells (6). IL-1β is produced as a 30kDa protein (pro-IL-1β); however, unlike pro-IL-1α, this form is not biologically active and requires cleavage in order to carry out its effector functions. Pro-IL-1β processing requires oligomerisation of inflammasome complexes, which can be assembled by multiple proteins (7, 8). Inflammasome activation facilitates activation of pro-caspase-1 to active caspase-1 and processing of its target proteins including IL-1β. The NLRP3 inflammasome has been best characterised and its activators include cholesterol, uric acid, viral particles and bacterial components and some vaccine adjuvants including alum (9, 10) (11, 12). Once the pro-form has been cleaved, a 17kDa active IL-1β protein can be secreted by the cell. It has also been shown that treatment of cells with inflammasome activators normally associated with IL-1β release can also lead to the secretion of IL-1α (13). Although caspase-1 activation, leading to IL-1β cleavage is commonly observed and this mechanism is well-elucidated, other mechanisms of inflammasome independent cleavage of pro-IL-1β can also occur (14). Caspase-1 independent IL-1β production has been mainly observed as a result of proteases such as elastase, proteinase-3, cathepsin G, granzyme A and chymase (15, 16). These enzymes cleave pro-IL-1β at sites close to that used by caspase-1. Pathways such as these provide an inflammatory mechanism different to the classical inflammasome dependent pathway.

Bile acids are amphipathic molecules synthesized from cholesterol in the liver (17). They are detergent molecules that facilitate intestinal absorption of lipids and nutrients. In cholestatic liver diseases, bile acids accumulate at high concentrations in the liver, resulting in hepatocyte injury, impaired liver function, fibrosis and cirrhosis. Primary bile acids in humans are Cholic acid (CA) and Chenodeoxycholic acid (CDCA). Primary bile acids are conjugated to glycine (most pronounced in humans) or taurine, secreted into the bile canaliculi, stored in the gall bladder, and released into the small intestine upon ingestion of a meal. Intestinal bacteria modify primary into secondary bile acids, such as Lithocholic acid (LCA) and Deoxycholic acid (DCA). Because the detergent characteristics of bile acids render them toxic for cells, their synthesis and transport are highly regulated via bile acid receptor signaling pathways. Bile acids have been shown to activate NF-κB and production of IL-8 *in vitro* (18, 19). In addition, bile acids have been implicated in various inflammatory diseases including gastrooesophageal reflux disease (GORD) and inflammatory bowel disease. In GORD, bile acid-containing refluxate is more damaging to the oesophagus than that in which bile acids are absent (20). These acids have been shown to have a number of effects on the squamous epithelial lining of the oesophagus in GORD, Barrett’s oesophagus and adenocarcinoma. Although bile acids induce pro-inflammatory signalling, their role in inducing innate immune cytokines and inflammation has not been fully explored to date. In this study we demonstrate that bile acids can stimulate IL-1α/β secretion by BMDCs and promote cell recruitment in an *in vivo* inflammation model. The data provide insight into the mechanisms through which bile acids can exert proinflammatory properties, particularly via their interactions with the innate immune system.

## 2 Methods

### 2.1 Materials

The following were used: Alhydrogel (Brenntag Biosector) referred to in the manuscript as ‘alum’, LPS (Enzo Life Sciences), RPMI 1640 medium (Biosera) supplemented with 8% inactivated FBS (Biosera), 2mM L-glutamine (Gibco) and 50 U/ml penicillin and 50 μg/ml streptomycin (Gibco), DCA and CDCA (Sigma-Aldrich), Z-YVAD-FMK (Bachem).

### 2.2 Animals

Female C57BL/6 (wild type, WT) mice were obtained from Harlan Olac (Bicester, Oxfordshire, UK). ASC^–/–^ breeding pairs from Genentech (San Francisco, CA, USA). NLRP3^–/–^ breeding pairs were provided by J. Tschopp (University of Lausanne, Switzerland). Mice were housed in the Bioresources Unit in Trinity College Dublin and were used at 10–16 weeks of age. Animals were maintained according to the regulations of the EU and the Irish Department of Health and all procedures performed were conducted under animal license number B100/3321 and were approved by the Trinity College Dublin Animal Research Ethics Committee (Ethical Approval Number 091210).

### 2.3 Primary dendritic cell isolation and culture

Bone marrow-derived dendritic cells (BMDCs) were generated as previously described (21). Briefly, bone marrow cells were isolated from tibias and femurs of mice. Ammonium chloride (0.88%) was used to lyse red blood cells. Cells were grown in complete RPMI supplemented with 20 ng/mL of granulocyte–macrophage colony-stimulating factor (GM-CSF) from the J588 myeloma cell line. On day 10, the loosely adherent cells were harvested and dendritic cells were plated at a density 6.25 x 10^5^cells/ml for cytokine analysis and cytotoxicity assay, or 5x10^5^ cells/ml for Western blot analysis. Cells were left overnight and were stimulated as indicated in figure legends.

### 2.4 Cytotoxicity assay

Cytotoxicity of bile acids was measured by flow cytometry using an LSR Fortessa (BD Biosciences). 1µl of 100µg/ml PI was added per 100µl of cells to each tube immediately prior to the tube being read by the flow cytometer. At least 10000 single cells were acquired from each sample. The percentage of PI positive cells was determined using FlowJo software (BD, Ashland, OR, USA).

### 2.5 Enzyme-linked immunosorbent assay

Enzyme-linked immunosorbent assay was performed on supernatants obtained from BMDCs to measure the cytokine secretion as a result of stimulation with bile acids. The cytokines were measured using commercially available kits for IL-1α (Biolegend) and IL-1β (R&D) according to manufacturers’ instructions and paired antibodies against IL-6 (BD Biosciences).

### 2.6 Western blot analysis

Processed and secreted IL-1β was visualized by Western blot analysis as described previously (22). Briefly, proteins from supernatants were isolated by methanol/chloroform precipitation; 500 μl of supernatant was mixed with 500 μl of methanol and 100 μl of chloroform and centrifuged at 13,000 rpm for 5 min. The supernatant was removed, and the protein pellet was washed with 500 μl methanol (13,000 rpm for 5 min). The protein pellet was air dried and resuspended in 20 μl of Laemmli buffer. Samples were loaded and separated on a 15% SDS-polyacrylamide gel and transferred to a PVDF membrane. The membrane was blocked for 1 hr at room temperature with 5% marvel in TBS/Tween 20 (0.1%). After washing with TBS/Tween, the membrane was probed with primary antibody (Santa Cruz; 1/1000 in 3% BSA) overnight at 4°C, washed three times with TBS/Tween and probed with HRP-conjugated secondary antibody (1/2000 in PBS) for 1 h at room temperature. The membrane was washed three times with TBS/Tween and HRP visualized by chemiluminescence.

### 2.7 *In vivo* cell recruitment experiment

WT mice were immunised intraperitoneally with PBS, 1mg DCA, 1mg CDCA or 1mg monosodium urate (MSU). After 24h, the mice were sacrificed. Peritoneal exudate cells (PECs) were collected by injecting 5ml PBS into the peritoneal cavity. Cells were washed with PBS, pelleted by centrifugation (400 g, 5 min, 4°C) and stained with Aqua LIVE/DEAD (0.25 μl, Invitrogen) for 25 min in the dark and on ice. After washing with PBS, cells were incubated with 50 μl of FACS buffer (2% FBS in PBS) mixed with anti-CD16/CD32 monoclonal antibodies (0.25 μg, clone 2.4G2, BD Pharmingen). Cells were then stained with the following fluorochrome-labelled antibodies: anti-CD11b (0.01 μg, clone M1/70; BD Pharmingen), anti-F4/80 (0.1 μg, clone BM8; eBioscience), anti-Gr-1 (0.1 μg, clone RB6-8C5; BD Pharmingen), for 15 min in the dark and on ice. Cells were washed twice and resuspended in 200μl of FACS buffer. Samples were acquired using a LSR Fortessa flow cytometer using FACSDiva software (BD Biosciences) and data were analysed using FlowJo software (BD, Ashland, OR, USA).

## 3 Results

**Figure 1:**
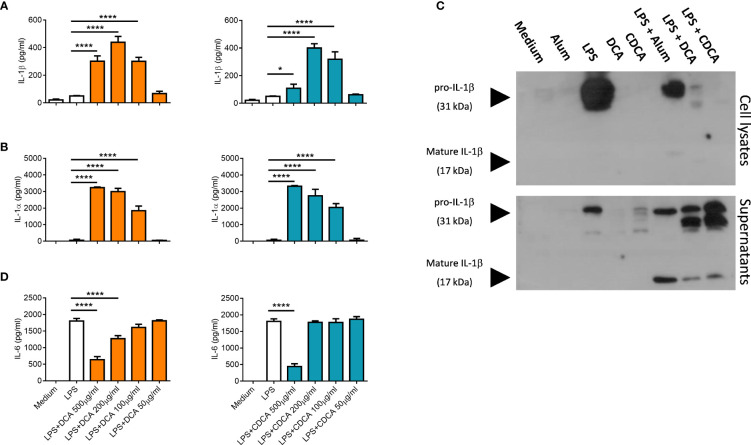
Bile acids induce IL-1α and IL-1β secretion from LPS-primed BMDCs. WT BMDCs (6.25x10^5^ cells/ml) were incubated in medium alone or were primed with 5ng/ml LPS for 3h before being stimulated with various concentrations (50µg/ml to 500µg/ml) of Deoxycholic acid (DCA) and Chenodeoxycholic acid (CDCA). Supernatants were collected 24h later and tested for IL-1α **(A)**, IL-1β **(B)** and IL-6 **(D)** by ELISA. Mean concentrations of cytokine (+/-SD) are shown for triplicate samples. Bile acids + LPS versus LPS alone, *p<0.05, ****p<0.0001 (One-way ANOVA). Data is representative of two independent experiments. **(C)** WT BMDCs (5x10^5^cells/ml) were incubated in medium or were primed with LPS for 3h before being stimulated with either DCA, CDCA (200μg/ml) or Alum (400 μg/ml) for 6hrs. Lysates and supernatants were resolved by SDS-PAGE and probed by Western blotting with anti-IL-1β antibody. Representative of two independent experiments.

**Figure 2:**
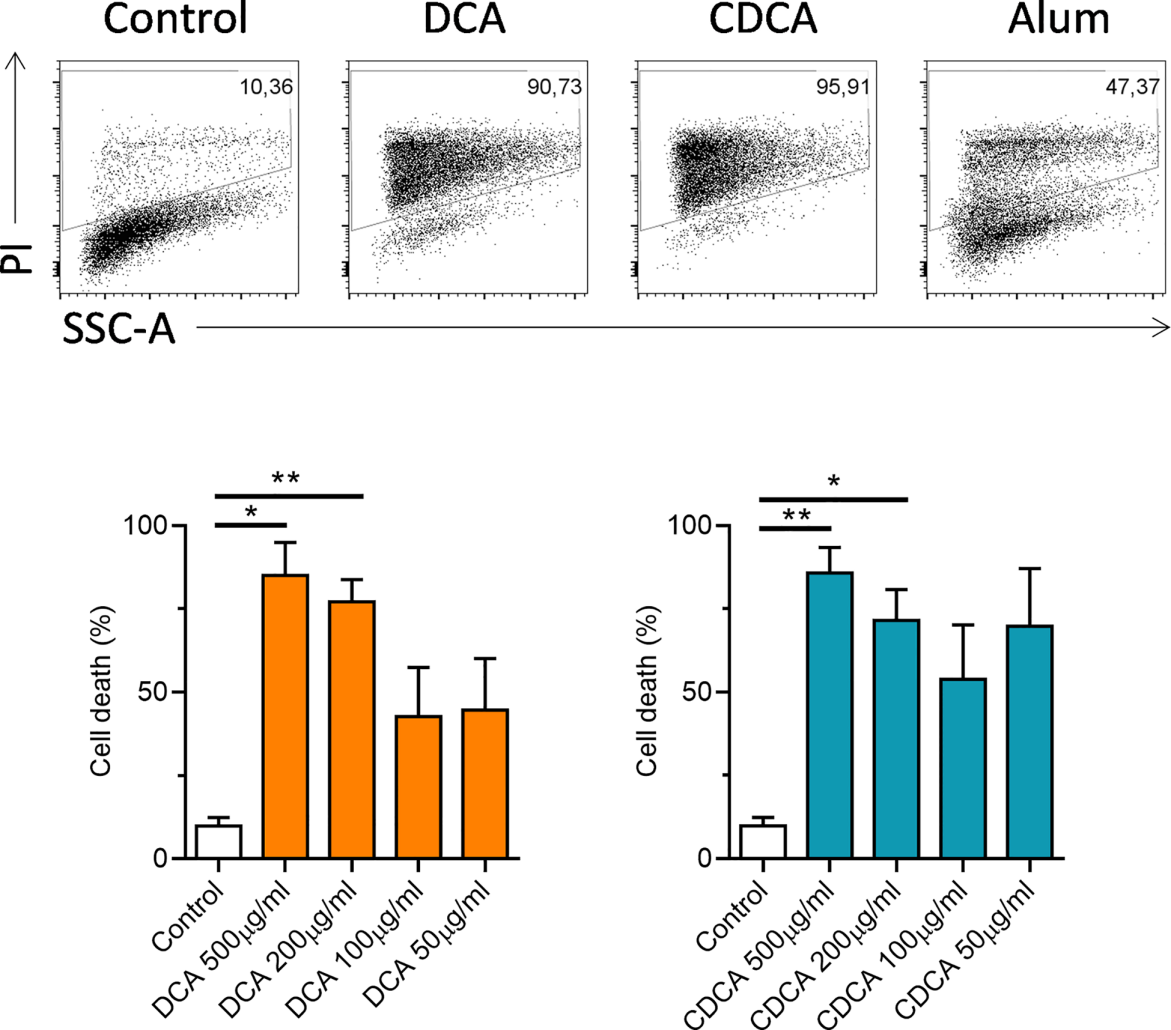
High concentrations of bile acids are toxic to BMDCs. WT BMDCs (6.25x10^5^/ml) were treated with the indicated concentrations of DCA or CDCA for 24h. Alum (400 µg/ml) was used as a positive control. Cells were stained with propidium iodide (PI) for each treatment and analysed by flow cytometry. Mean percentage of PI positive cells (+SEM) are shown for DCA or CDCA. Stimulation with bile acids versus medium *<p0.05, **p<0.01 (One-way ANOVA). Data from four independent experiments.

IL-1α and IL-1β are a pro-inflammatory cytokines, that are secreted by activated dendritic cells (23). The ability of crystalline and particulate materials such as alum to mediate IL-1 secretion in dendritic cells has been established (24). In this study we investigated whether bile acids had the potential to drive IL-1 secretion. BMDCs were primed with LPS to stimulate intracellular pro-IL-1α and pro-IL-1β accumulation, then bile acids were added to assess if they can activate IL-1 release. Both bile acids, DCA and CDCA promote IL-1α and IL-1β secretion from BMDCs primed with LPS in a concentration-dependent manner (**Figures 1A, B**). DCA and CDCA did not induce IL-1α and IL-1β secretion at the lower (50µg/ml) end of the concentration range tested. For both DCA and CDCA, 200µg/ml of bile acid induced the highest level of IL-1β while at a concentration of 500µg/ml production of IL-1β was attenuated, particularly by CDCA. IL-1α release was highest in BMDCs treated with 500μg/ml CDCA or DCA. Next, Western blotting was used to determine if the bile acids promoted cleavage of pro-IL-1β (30kDa) to its bioactive form (17kDa) (**Figure 1C**). The disappearance of a 30kDa band from the lysates and the appearance of a 17kDa band in the supernatants in LPS-primed samples stimulated with 200 µg/ml DCA and 200 µg/ml CDCA indicates that these bile acids provide a signal which induces the cleavage of pro-IL-1β into its active, secreted form. This cleavage is clearly observed when LPS primed BMDCs treated with alum, which induces pro-IL-1β processing, are compared to those cells stimulated with LPS alone. Furthermore, high concentrations of DCA or CDCA (500μg/ml) result in decreased IL-6 production in LPS-primed BMDCs (**Figure 1D**). This may be due to toxicity of bile acids. Thus, to assess the cytotoxicity of the DCA and CDCA, treated cells were stained with propidium iodide (PI). PI staining showed that high concentrations of both DCA and CDCA are toxic to dendritic cells. Exposure of BMDCs to bile acid concentrations of 500µg/ml and 200µg/ml resulted in greater than 75% PI-positive cells. Both DCA and CDCA showed a similar pattern of cytotoxicity across the concentration range (**Figure 2**).

**Figure 3:**
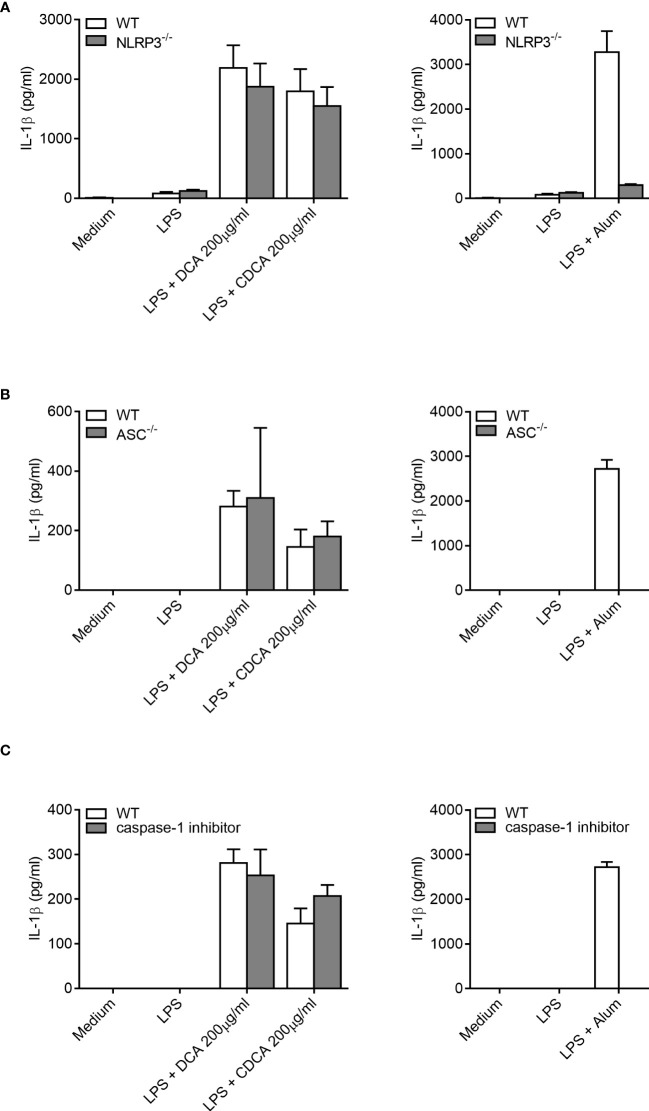
IL-1β secretion by BMDCs in response to bile acids is not dependent on ASC, the NLRP3 inflammasome or caspase-1. BMDCs (6.25x10^5^ cells/ml) from either wild type (WT), NLRP3^-/-^ **(A)**, ASC^-/-^ mice **(B)** or pre-treated with caspase-1 inhibitor Z-YVAD-FMK (10μM) **(C)** were primed with 5ng/ml LPS for 3h before being stimulated with 200µg/ml of DCA, 200µg/ml of CDCA or 1000µg/ml Alum. Supernatants were collected 24h later and tested for IL-1β by ELISA. Results are mean cytokine concentration (+/-SD) for triplicate samples. Representative of three independent experiments.

Next, the mechanism of IL-1β processing was investigated. Inflammasomes are multi-protein complexes that forms in the cell in response to inflammatory signals and facilitate the cleavage of pro-IL-1β to IL-1β. Therefore, inflammasome activation may be implicated in the mechanism of IL-1β secretion in response to bile acids. The NLRP3 inflammasome, a complex of Nod like receptor (NLR) protein 3, (NLRP3) apoptosis-associated speck-like protein containing a CARD (ASC) and Caspase-1, has been shown to be crucial for IL-1β processing *in vitro* following various stimuli (24). Using BMDCs from NLRP3^-/-^, and ASC^-/-^ mice, we investigated the role of the NLRP3 inflammasome in IL-1β release from LPS-primed BMDCs stimulated with DCA or CDCA (**Figures 3A, B**). LPS-primed BMDCs from either wild-type or knockout mice were treated with 200 μg/ml bile acid overnight and IL-1β levels were measured (**Figures 3A, B**). Alum was used as a control as this particulate adjuvant induces NLRP3 inflammasome activation *in vitro.* It has been shown that cells deficient in NLRP3, ASC or caspase-1 do not secrete IL-1β following priming with LPS and subsequent activation with alum (24).

**Figure 4:**
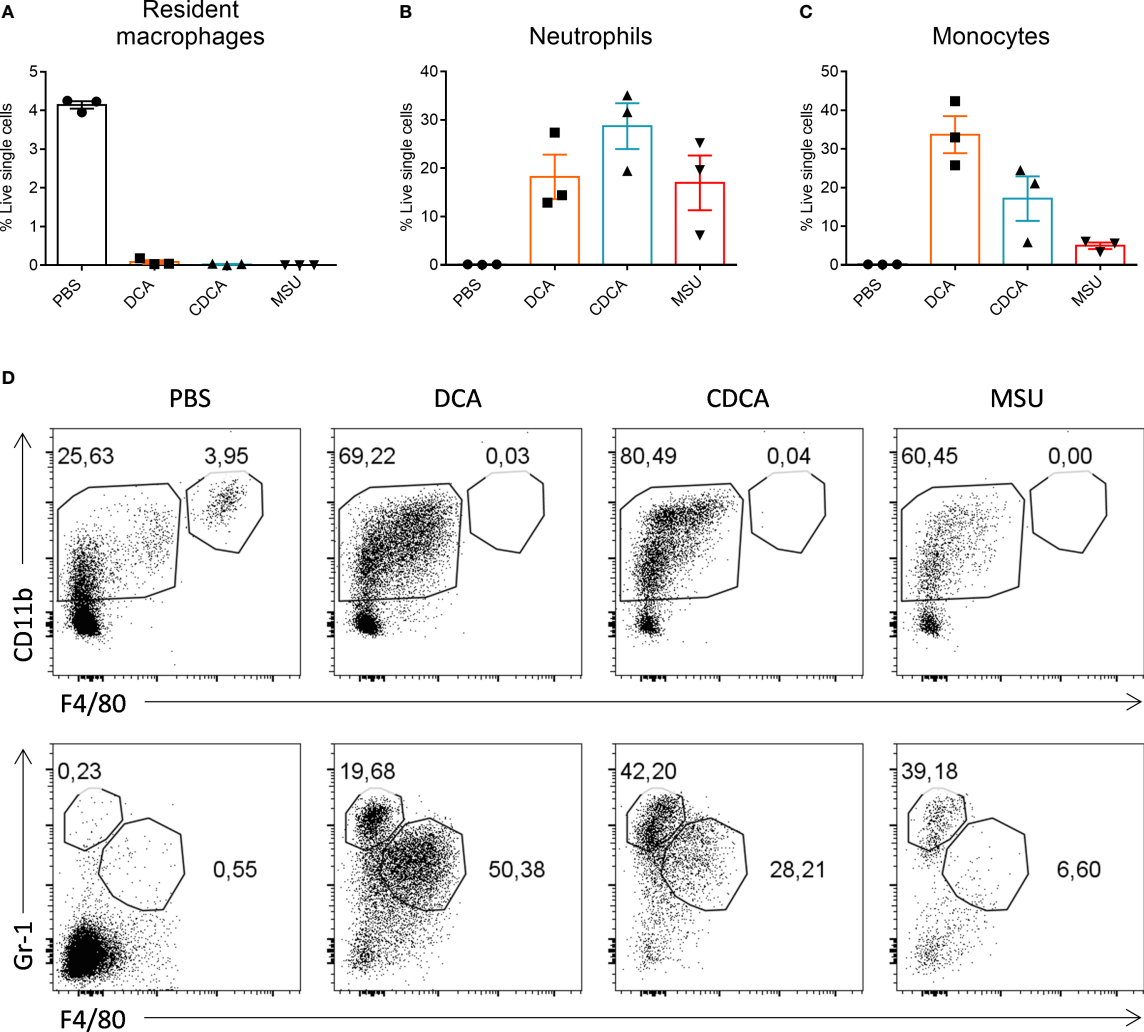
Bile acids induce neutrophil and monocyte recruitment *in vivo*. Bile acids induce neutrophil and monocyte recruitment *in vivo*. WT mice were injected intraperitoneally with PBS, 1000µg of DCA, CDCA or MSU. 3 mice were used for each treatment. After 24h lavage was performed by washing the peritoneum with PBS and collecting the fluid. Cell populations were analysed by flow cytometry for the presence of resident macrophages **(A)**, neutrophils **(B)** and monocytes **(C)**. Mean percentage cells (+/- SEM) is shown for each treatment group. **(D)** Representative gating for CD11b^+^F4/80^+^ resident macrophages (top panel); CD11b^+^F4/80^-^Gr^-^1^+^ neutrophils and CD11b^+^F4/80^+^Gr^-^1^+^ monocytes (bottom panel) in each treatment group.

When compared to WT BMDCs, cells from the NLRP3^-/-^and ASC^-/-^ mice secreted similar levels of IL-1β when stimulated with LPS with DCA or CDCA (**Figures 3A, B**). In contrast, Alum-induced Il-1β secretion was dependent on NLRP3 and ASC. In addition, IL-1β secretion was compared between WT BMDCs treated with bile acid in the presence or absence of the caspase-1 inhibitor Z-YVAD-FMK. No difference was observed in the secretion of IL-1β between inhibitor treated and untreated cells following DCA or CDCA stimulation in LPS primed BMDCs (**Figure 3C**). In contrast, Alum-induced IL-1β secretion was dependent on caspase-1. As DCA and CDCA induced secretion of the proinflammatory cytokine IL-1β from BMDCs *in vitro*, their ability to induce inflammation *in vivo* was determined. We utilised a mouse model of intraperitoneal injection as it is an established model to determine local innate immune response. It has been extensively used to analyze the immune response to adjuvants, MSU crystals, bacterial infections, and TLR ligands (24–29). Recruitment of cells to the site of injection was compared between control (PBS), DCA, CDCA and Monosodium urate crystals (MSU) treatments. MSU was used as a positive control as it induces neutrophil infiltration in an IL-1 dependent manner (10, 28). Firstly we compared resident macrophages which disappear from the peritoneum during inflammation due to cell death or adhesion to mesothelium (29, 30). Indeed, we observed that resident macrophages were present in mice from the PBS group as being CD11b^+^ F4/80^+^. However, this population could no longer be seen in those mice that had been injected with DCA, CDCA or MSU crystals (**Figures 4A, D**). In mice injected with DCA, CDCA or MSU, a new population of CD11b^+^ Gr-1high F4/80^-^ neutrophils was recruited (**Figures 4B, D**). Furthermore, a population of monocytes also appeared in those mice that had been injected with DCA, CDCA or MSU (**Figures 4C, D**). Overall, these results highlight that DCA and CDCA induce an inflammatory response *in vivo*.

## 4 Discussion

Bile acids have classically been recognised as molecules that are produced by the liver and circulate between the liver and intestine where they aid in the solubilisation and absorption of dietary fat. More recently it has become apparent that these molecules play important roles in inflammation and metabolism signalling through receptors that include FXR and TGR5 [reviewed in (17, 31)]. Bile acid-induced activation of FXR can regulate metabolism of lipids, glucose and bile acids themselves (32). Their stimulation of TGR5 can modulate energy expenditure and glucose tolerance (33, 34). Bile acids are pro-inflammatory and cytotoxic when dysregulated, an example being cholestatic liver diseases where accumulation of bile acids leads to chronic inflammation, hepatocellular necrosis, and liver failure (35, 36). In our study we investigated the impact of a primary bile acid, CDCA and a secondary bile acid DCA on the inflammatory response in dendritic cells as these cells have been previously shown to be involved in these inflammatory diseases. Importantly, other bile such as cholic acid, glycocholic acid, lithocholic acid, or taurolithocholic acid might also exhibit similar properties.

Here we show that bile acids can induce the production of key inflammatory cytokines IL-1α and IL-1β by BMDCs. The enhancement of IL-1β production in LPS-primed BMDCs was demonstrated for both deoxycholic acid (DCA) and chenodeoxycholic acid (CDCA) over a range of concentrations. Importantly, we show that IL-1β is released in its bioactive form (17kDa), which indicates that bile acids induce a specific pathway to cleave pro-IL-1β.

IL-1β is a potent inflammatory cytokine which causes changes in vascular endothelial cells allowing them to recruit immune cells as well releasing chemotactic cytokines such as IL-8; these processes are central to many inflammatory states. Inflammation is associated with cell death and release of DAMPs (1). In our study we show that bile acids induce release of IL-1α which can be facilitated by cell death. IL-1α does not require processing and is active in its pro-form (37). We also show that at concentrations of 200-500μg/ml, DCA and CDCA are cytotoxic which can drive release of danger-associated molecular patterns and also impact inflammatory response to these bile acids. We investigated cell death via propidium iodide (PI) staining which is routinely used to analyse membrane permeabilization in cells and indicates cell death. However, we cannot rule out that bile acids induce specific cell death pathways associated with lytic cell death such as pyroptosis, necroptosis or PANoptosis in BMDCs (38).

Bile acids can induce cell death via a number of pathways in various cells. Cytotoxicity of bile acids can be cell type specific and also related to factors such as hydrophobicity and conjugation status of the bile acids. Their effects can be ‘non-specific’ e.g. disruption of lipid bi-layers via their detergent activity or mediated *via* extrinsic activation of apoptosis through multiple receptors and intrinsically through mitochondrial perturbations and oxidative stress and oxidative DNA damage (36, 39, 40). It would be interesting to investigate in future studies what type of cell death is induced by bile acids in various immune cells.

Importantly, bile acids can induce cleavage of pro-IL-1β to IL-1β, which highlights the fact that aside from their cytotoxicity, bile acids can activate specific cellular pathways. The current study has clearly shown that bile acid-induced IL-1β release is independent from NLRP3 inflammasome activation in BMDCs. Although the NLRP3 inflammasome is the best studied of the inflammasomes other members of this family have also been implicated in IL-1β activation. Other inflammasomes which use the adaptor protein ASC (apoptosis-associated speck-like protein containing a caspase recruitment domain) include the NLRP1, NLRC4 and AIM2 inflammasomes (7, 8, 41). When compared with WT BMDCs no difference in levels of IL-1β were observed after stimulation with bile acids in ASC-deficient cells indicating that this molecule, and therefore any ASC dependent inflammasome, is not involved in the pathway for inflammation caused by bile acids. Furthermore, incubation of the BMDCs with a caspase-1 specific inhibitor prior to stimulation with bile acids did not reduce IL-1β secretion, supporting evidence that bile acid induced IL-1β secretion is not caspase-1 mediated. Reports on the role of the NLRP3 inflammasome in cellular responses to bile acids are somewhat contradictory. Guo et al. demonstrated that the bile acids lithocholic acid or taurolithocholic acid inhibited IL-1β production by TGR5-cAMP-PKA-mediated phosphorylation (and subsequent ubiquitination) of the NLRP3 inflammasome in bone-marrow-derived macrophages (BMDMs) (42). Interestingly, BMDMs deficient in Tgr5 or Fxr have impaired inflammasome activation during bacterial infection (43). However, these cells were not studied in the context of direct bile acid activation or any classical NLRP3 activators.

In addition, TGR5 inhibiton attenuated alum-induced peritoneal inflammation. However, in this study bile acids were not used in an *in vivo* peritonitis model, thus a direct inhibitory effect of bile acids on inflammation was not described. Interestingly, Ichikawa et al. have shown that monocytes treated with GM-CSF and IL-4 rapidly lose expression of TGR5 at mRNA and protein level during the differentiation process with no expression in BMDCs (44). Therefore, while it has been shown that Tgr5 plays a role in IL-1β processing in macrophages, its role in dendritic cells needs to be validated as its involvement may be cell-specific.

On the other hand, Gong et al. demonstrate that CDCA promotes IL-1β secretion in the macrophage cell line J774A.1. It has been demonstrated that silencing Nlrp3 decreases IL-1β production (45). They also showed that a caspase-1 inhibitor decreased levels of IL-1β and ameliorated liver fibrosis in an *in vivo* bile duct ligation model of cholestasis. The authors used higher concentration of LPS (1μg/ml) and lower concentrations of CDCA (50-100μM, approximately 20-40μg/ml) compared to our study. The high concentration of LPS might induce higher levels of pro-IL-1β in cells and even lower concentrations of CDCA might lead to IL-1β release by cells. The authors of this publication suggest that differences in priming of cells in their model and a demonstrated role for the EGFR pathway in bile-acid induced NLRP3 activation may account for the opposite effect of bile acid on NLRP3 activity seen in their model. Zhao et al. studied the effect of DCA in the DSS-induced murine model of colitis (46). Again, using the same experimental set up they demonstrated that DCA induced IL-1β secretion in J774A.1 cells and that silencing Nlrp3 decreases IL-1β production *in vitro*. Interestingly, in this publication it was shown that intraperitoneal injection of DCA worsened DSS-induced colitis and this effect was abrogated by addition of caspase-1 inhibitor or macrophage depletion. Thus it suggests that macrophage-derived IL-1β induced by DCA has a pathogenic contribution to disease severity. We have clearly shown that both DCA and CDCA induce IL-1β secretion in BMDCs, however using NLRP3-deficient cells we have demonstrated that IL-1β secretion is NLRP3 inflammasome-independent. There are numerous examples of inflammasome-independent production of IL-1β described in the literature. In a mouse model of osteomyelitis (*Pstpip2* ^cmo^ mice), the uncontrolled IL-1β production driving bone inflammation was independent of caspase-1 and NLRP3 (47). Interestingly, neutrophils from these mice secreted elevated levels of IL-1β in response to ATP, silica and *Pseudomonas aeruginosa* compared to neutrophils from WT mice with aberrant IL-1β production being sensitive to serine protease inhibition (48). Similarly, diesel exhaust particle-induced pulmonary inflammation is mediated by the IL-1β/IL-1R1 axis but is NLRP3/Caspase-1-independent (49).

Overall, we demonstrate that bile acids can induce the production of IL-1α and IL-1β in BMDCs with IL-1 production being ASC/NLRP3/caspase-1-independent. We also demonstrate direct effect of bile acids on cell recruitment into the site of injection. These findings are illustrated in a schematic diagram in **Supplementary Figure 1**. Both, DCA and CDCA promote neutrophil and monocyte infiltration *in vivo* underlining the physiological importance of their pro-inflammatory activity.IL-1α and IL-1β plays an important role in regulating innate and adaptive immune responses. Aberrant production of these cytokines is associated with sterile inflammatory conditions such as atherosclerosis, type 2 diabetes and gout (50). The demonstration that bile acids can promote IL-1α and IL-1β secretion suggests that this process may have an important role to play in pathology of many diseases, including those of the liver and gastrointestinal tract. It has been shown that dendritic cells (DCs) are present in the lamina propria of individuals with Barrett’s oesophagus and adenocarcinoma (51). We postulate that these cells, via their interaction with bile acids and production of IL-1, may play a role in the chronic inflammation associated with these diseases. Overall, our results highlight that bile acids have diverse pro-inflammatory properties which impact on innate immunity and subsequently can modulate various disease outcomes.

## Data availability statement

The original contributions presented in the study are included in the article/**Supplementary Material**. Further inquiries can be directed to the corresponding author.

## Ethics statement

The animal study was approved by Trinity College Dublin Animal Research Ethics Committee. The study was conducted in accordance with the local legislation and institutional requirements.

## Author contributions

EO: Formal Analysis, Investigation, Methodology, Writing – review & editing. ECO: Formal Analysis, Investigation, Writing – review & editing. MF: Formal Analysis, Investigation, Writing – review & editing. EL: Conceptualization, Methodology, Resources, Supervision, Writing – review & editing. AL: Conceptualization, Resources, Supervision, Writing – original draft, Writing – review & editing.
