# Supplementary figures and images for: Bile acids induce IL-1α and drive NLRP3 inflammasome-independent production of IL-1β in murine dendritic cells
Source: Front Immunol. 2023 Nov 27;14:1285357. doi: 10.3389/fimmu.2023.1285357 (PMC10711081; doi:10.3389/fimmu.2023.1285357)

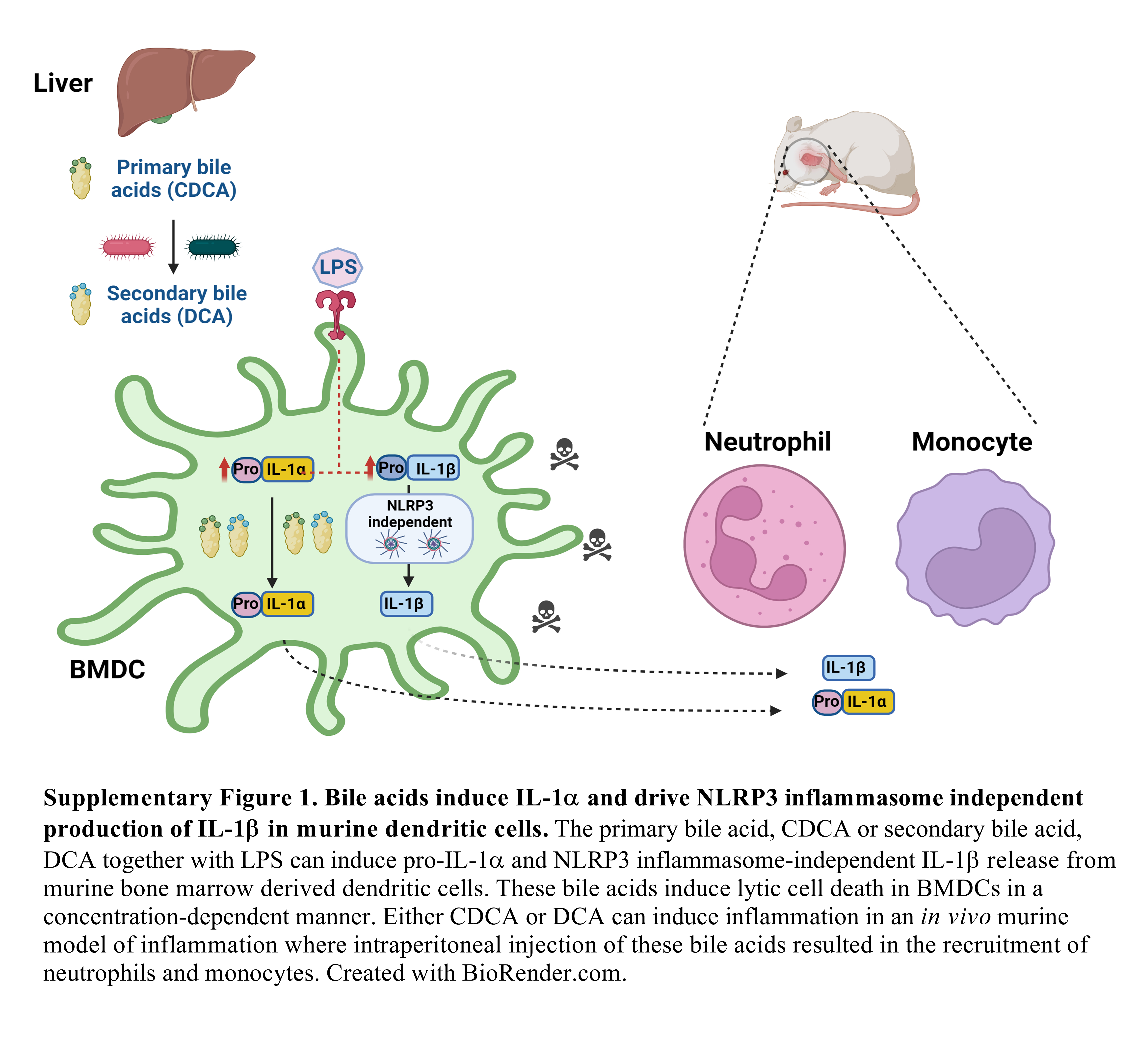

Supplement: Supplementary file 1 [file Image_1.tif]
